# Lenvatinib combined with anti-PD-1 antibodies plus transcatheter arterial chemoembolization for neoadjuvant treatment of resectable hepatocellular carcinoma with high risk of recurrence: A multicenter retrospective study

**DOI:** 10.3389/fonc.2022.985380

**Published:** 2022-09-21

**Authors:** Jun-Yi Wu, Jia-Yi Wu, Yi-Nan Li, Fu-Nan Qiu, Song-Qiang Zhou, Zhen-Yu Yin, Yu-Feng Chen, Bin Li, Jian-Yin Zhou, Mao-Lin Yan

**Affiliations:** ^1^ Shengli Clinical Medical College of Fujian Medical University, Fuzhou, China; ^2^ Department of Hepatobiliary Pancreatic Surgery, Fujian Provincial Hospital, Fuzhou, China; ^3^ Department of Hepatobiliary Surgery, Xiamen Traditional Chinese Medical Hospital, Xiamen, China; ^4^ Department of Hepatobiliary Surgery, The Zhangzhou Affiliated Hospital of Fujian Medical University, Zhangzhou, China; ^5^ Department of Hepato-Biliary-Pancreatic and Vascular Surgery, The First Affiliated Hospital of Xiamen University, Xiamen, China; ^6^ Department of Hepatobiliary Surgery, Zhongshan Hospital of Xiamen University, Xiamen, China

**Keywords:** hepatocellular carcinoma, neoadjuvant treatment, triple therapy, disease-free survival (DFS), overall survival (OS)

## Abstract

**Background:**

Early recurrence is common after surgical resection (SR) for hepatocellular carcinoma (HCC) with high risk of recurrence and is associated with poor prognosis. The combinations of lenvatinib (LEN), anti-PD-1 antibodies (PD-1) and transcatheter arterial chemoembolization (TACE) (triple therapy) has shown better trend in tumor response and survival outcomes on unresectable HCC. It is unknown whether triple therapy for neoadjuvant treatment of resectable HCC with high risk of recurrence is effective. This article aimed to compare the outcomes of surgery alone and neoadjuvant combination treatment with triple therapy before SR in patients with HCC with high risk of recurrence.

**Methods:**

A retrospective study was conducted on patients diagnosed with HCC with high risk of recurrence who received treatment with or without triple therapy. The records of 24 patients in the triple therapy group and 76 patients in the surgery-alone group were analyzed. Propensity score matching (PSM) was performed to minimize the influence of potential confounders.

**Results:**

One hundred patients were enrolled. In the triple therapy group, 8 (33.3%) and 12 (50.0%) patients had complete and partial responses, respectively, as assessed by an investigator. Before PSM, the overall survival (OS) rates for the triple therapy group at 6, 12, 18, and 24 months were 100.0%, 100.0%, 100.0%, and 85.7%, respectively, compared with corresponding 92.1%, 73.7%, 53.9%, and 48.7% for the surgery-alone group (P<0.001). The disease-free survival (DFS) rates were 82.2%, 66.95%, 48.8%, and 48.8% for the triple therapy and 41.92%, 28.34%, 27.05%, and 22.99% for the surgery-alone group (P=0.003). After PSM, DFS and OS were significantly longer in the triple therapy group than in the surgery-alone group (DFS, p=0.019; OS, p=0.003).

**Conclusions:**

Neoadjuvant combination treatment before SR had a high rate of tumor response and provided significantly better postoperative survival outcomes than surgery alone in patients with HCC with high risk of recurrence.

## Background

Hepatocellular carcinoma (HCC) is one of the most commonly diagnosed cancers and a leading cause of cancer-related death worldwide (1, 2). Surgical resection (SR) is the best choice for curative treatment of patients with HCC who have good functional liver reserves (3, 4). However, the inclusion criteria for selecting patients with HCC for SR remain controversial. In Western guidelines, resection is restricted to patients with early-stage HCC, which is based on the Barcelona Clinic Liver Cancer (BCLC) system classification (5). However, in China or Southeast Asia, it has adopted a more liberal application of SR for higher-burden HCC, including patients with large tumor size, tumor multiplicity, and portal vein tumor thrombus (PVTT) (6, 7). Moreover, many studies have indicated that patients with more advanced HCC would also benefit from SR as first-line therapy compared with nonoperative treatments, such as transcatheter arterial chemoembolization (TACE) and systemic therapy (8–11). However, SR for these patients with higher-burden HCC was associated with high rates of early recurrence and worse survival rates (12, 13). Therefore, effective treatments for patients with higher-burden HCC are urgently needed to reduce recurrence after SR and improve prognosis.

Presently, to reduce the tumor burden and improve the prognosis in patients with high risk of recurrence, neoadjuvant therapies have been performed before SR for many commonly occurring cancers, such as breast cancer, colon cancer, and esophageal cancer (14–16). Unlike these commonly occurring cancers, the use of neoadjuvant therapies for HCC with high risk of recurrence remains insufficiently effective (17). Several locoregional therapies, including TACE, have been used as neoadjuvant therapies for HCC with high tumor burden, but the clinical benefit was unsatisfactory (18–20). Until recently, multiple combinations of systemic therapies, including tyrosine kinase inhibitors (TKIs) and immune checkpoint inhibitors (ICIs), have shown the potential to improve the prognosis of advanced HCC (21–23). Based on recent studies on combination therapy with several systemic therapeutic agents in advanced HCC, they might be potential candidates for neoadjuvant treatment before SR in patients with HCC with high risk of recurrence.

In our previous study, a combination of lenvatinib (LEN), anti-PD-1 antibodies (PD-1), and TACE (triple therapy) showed a high rate of tumor response and converted resection in patients with unresectable HCC (uHCC) with manageable toxicity (24). However, the effect of triple therapy as neoadjuvant treatment of resectable HCC with high risk of recurrence is still unknown. This study aimed to investigate the safety and clinical efficacy of triple therapy in patients with HCC with high risk of recurrence.

## Methods

### Patients

The present study retrospectively reviewed patients with HCC patients with high risk of recurrence who received triple therapy (LEN+PD-1+TACE) before SR or underwent SR alone between November 2018 and December 2020 at four high-volume institutions: Fujian Provincial Hospital, Zhongshan Hospital of Xiamen University, First Affiliated Hospital of Xiamen University, and Zhangzhou Affiliated Hospital of Fujian Medical University. Baseline data, including preoperative, operative, and postoperative demographic details and outcomes, were retrospectively collected. This study was approved by the research ethics committee of each institution. All patients or their guardians provided written informed consent prior to enrolment.

The diagnosis of liver cirrhosis was based on the imaging of computerized tomography (CT) or magnetic resonance imaging (MRI). The diagnosis of HCC was based on biopsy or clinicoradiological criteria according to the guidelines proposed by the European Association for the Study of Liver (25). All HCC diagnoses were pathologically confirmed by two experienced pathologists after SR. In our study, HCC with high risk of recurrence was defined as follows: (1) HCC with Cheng’s type II PVTT (PVTT involving the left- or right-side branch) (26), (2) single huge HCC (tumor size >10 cm) and tumors adjacent to the major vascular structures (including the main portal branches, main trunks of the hepatic veins, and inferior vena cava) leading to narrow-margin hepatectomy (resection margin <1 cm), and (3) unilobar multifocal disease (>3 tumors and one tumor >5 cm).

The key inclusion criteria were as follows: (1) age 18–70 years with good operative tolerance; (2) resectable primary HCC; (3) HCC with high risk of recurrence; (4) no distant metastasis; (5) the future liver remnant (FLR) of HCC patients with or without liver cirrhosis were ≥ 40% and 30% of the total liver volume respectively; and (6) Eastern Cooperative Oncology Group performance status (ECOG PS) score of 0–1. The exclusion criteria were as follows: (1) combined HCC and cholangiocarcinoma; (2) other serious malignant diseases; (3) Child-Pugh class C; (4) PVTT involving the bilateral or main trunk of the portal vein; (5) death of other disease-related causes; (6) any other previous antitumor treatment, such as radiofrequency ablation, radiotherapy, systemic therapy, and chemotherapy before SR; and (7) incomplete data.

### Neoadjuvant triple therapy (LEN + PD-1 + TACE) and evaluation of response or toxicity

In the triple therapy group, the treatment period of neoadjuvant triple therapy was decided to 3 cycles in advance. Patients received LEN at a dose of 12 mg for body weight ≥60 kg or 8 mg for body weight <60 kg orally daily, and PD-1 at a dose of 200 mg sintilimab, 200 mg camrelizumab, 200 mg tislelizumab, 200 mg pembrolizumab, or toripalimab 240 mg intravenously every 3 weeks. TACE was performed within 7 days of diagnosis. Depending on the size, location, and arterial supply of the tumor, a mixture of iodized oil and pirarubicin was injected into the selected tumor artery through the microcatheter used for chemoembolization. Then, gelatin sponge particles were advanced toward the tumor-feeding arteries for selective embolization. Patients underwent a restaging scan and surgical evaluation every 4 weeks *via* the assessment of alpha-fetoprotein (AFP) levels and CT or MRI. LEN and PD-1 were discontinued for three days before and after TACE. SR was performed at least 3 weeks after the last dose of PD-1 and 1 week after the last dose of LEN. All patients with active HBV infection received oral antiviral treatment.

Tumors were assessed using modified criteria (mRECIST) both by the investigator and blinded independent central review (BICR) to evaluate the therapeutic effects of neoadjuvant triple therapy on primary HCC based on measurable diameter and arterial enhancement *via* enhanced CT or MRI. The categories of tumor response were as follows: complete response (CR), partial response (PR), stable disease (SD), and progressive disease (PD) using mRECIST. The objective response rate (ORR) was defined as the proportion of patients with CR or PR. The disease control rate was defined as CR, PR, and SD. Images were evaluated by two experienced readers, a radiologist and a surgeon, in consensus.

In the triple therapy group, after followed by 3 cycles of neoadjuvant triple therapy, the curative effect of HCC patients with high risk of recurrence was evaluated as SD, then these HCC patients would continue to be treated with neoadjuvant triple therapy until they were evaluated as PR or PD or neoadjuvant triple therapy was more than 12 months. After 3 cycles of neoadjuvant triple therapy, TACE was performed if there was an obvious hepatic arterial blood supply to HCC every 4–6 weeks according to the CT or MRI results. In the triple therapy group, when patients were evaluated as PD which was considered unsuitable for surgery, they were treated with nonsurgical therapy with regorafenib.

Pathologic CR was defined as complete absence of viable tumor cells, while major pathologic response was defined as ≤10% of viable tumor cells in the postoperative pathology. Toxicities were evaluated according to the Common Terminology Criteria for Adverse Events version 5.0.

### Surgical procedure

In the surgery alone group, surgery was planned within 7 days of diagnosis. In the triple therapy group, patients with HCC were treated with triple therapy immediately within 7 days at the time of diagnosis and re-evaluated every 4 weeks after neoadjuvant triple therapy. In the triple therapy group, patients with HCC who were eligible for SR underwent definitive SR. When the tumor response was assessed as SD, surgery was planned after 3 months if the patients did not develop a contraindication to surgery. R0 resection was defined as histologically negative specimen margins, R1 as histologically positive margins, and R2 as macroscopically positive margins.

### Follow-up

The primary endpoint was overall survival (OS), which was defined as the time from initial diagnosis to tumor-related death. One of the secondary outcomes was disease-free survival (DFS), which was defined as the time from the initial surgery to the time when a recurrent tumor was first diagnosed. The other secondary endpoints were ORR in the triple therapy group and the rates of microvascular invasion (MVI) and R0 resection after SR.

All patients were treated with TACE 4 weeks after SR. In the triple therapy group, the patients continued to receive LEN plus PD-1 for 4–12 months after SR. Follow-up was performed every 3 months with assessment of AFP levels, liver function, and double-phase helical CT or MRI. Recurrence was managed with multimodality treatments, including SR, radiofrequency ablation, TACE, or systemic therapy, based on the recurrence pattern and functional liver reserves. All patients were followed until death or the study end date of April 2022.

### Statistical analysis

Statistical analyses were performed using SPSS software (version 17.0; SPSS Inc., Chicago, IL, USA), R3.1.2 software (Institute for Statistics and Mathematics, Vienna, Austria) and GraphPad Prism software (version 8.0; GraphPad Prism Software Inc., San Diego, CA, USA). Continuous data are presented as mean (s.d.) and analyzed using independent t-test. Categorical data were compared using the chi-squared test or Fisher’s exact test. OS and DFS rates were calculated using Kaplan–Meier estimates and compared using the log-rank test.

Propensity score matching (PSM) analysis was performed to reduce possible selection bias using a 2:1 matching method using the package (MatchIt) *via* R3.1.2 software. Sex, age, hepatitis B surface antigen (HBsAg), liver cirrhosis, serum AFP, protein induced by vitamin K absence-II (PIVKA-II), tumor number, tumor diameter, ECOG PS, PVTT, and total bilirubin (Tbil), albumin (ALB), and alanine aminotransferase (ALT) levels were entered into the PSM.

## Results

### Patient characteristics

**Figure 1:**
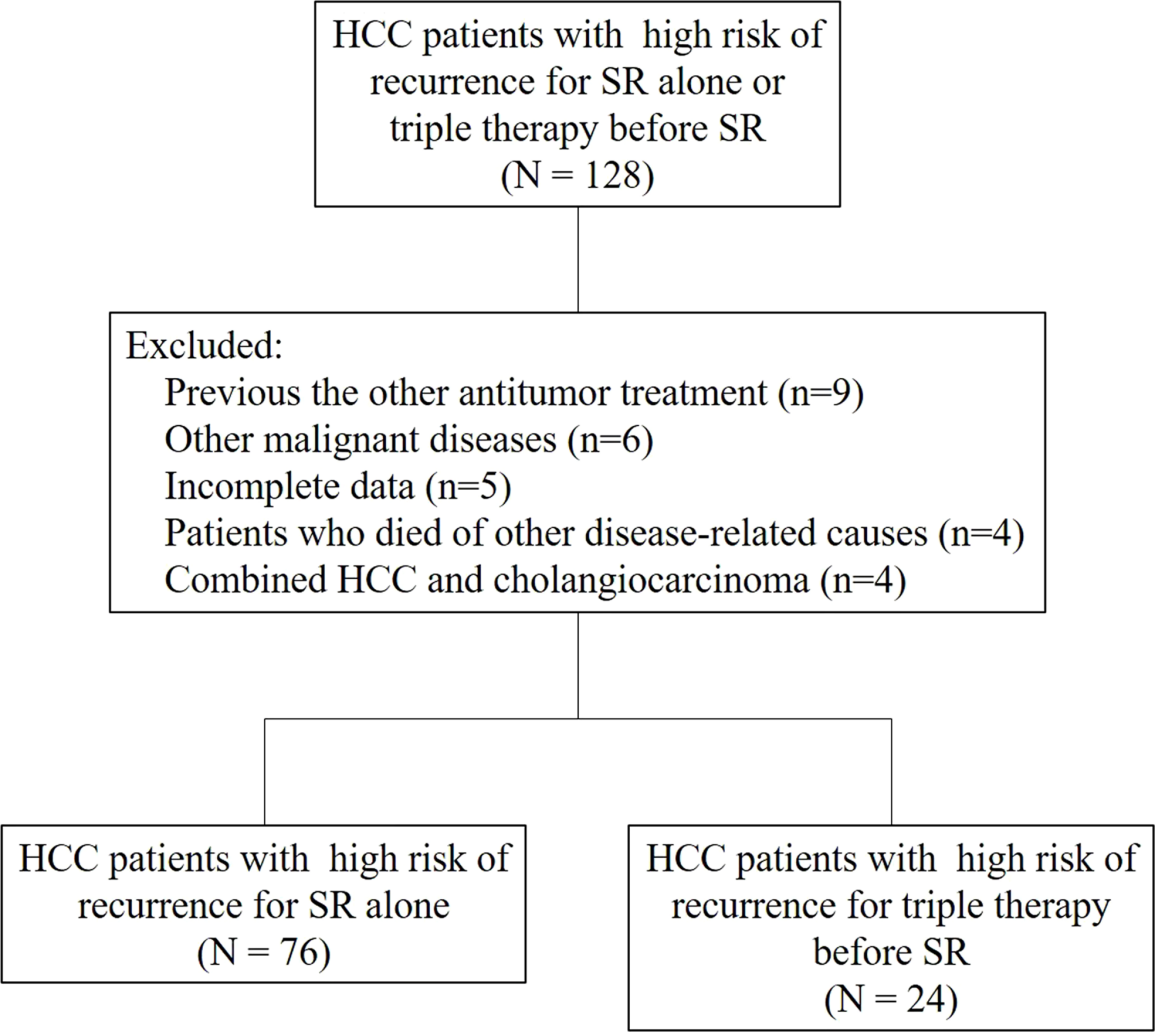
Flow diagram of the study selection process.

The baseline demographics and characteristics of the patients with HCC are shown in **Table 1**. A total of 100 patients with HCC with high risk of recurrence were included in our analysis, including 24 patients with HCC in the triple therapy group and 76 in the surgery-alone group (**Figure 1**). These patients were obtained from the Fujian Provincial Hospital (n=46), Zhongshan Hospital of Xiamen University (n=17), First Affiliated Hospital of Xiamen University (n=30), and Zhangzhou Affiliated Hospital of Fujian Medical University(n=7). Of the total patients, there were no significant differences in sex, age, HBsAg, liver cirrhosis, Child-Pugh class, AFP, PIVKA-II, tumor number, tumor diameter, ECOG PS, PVTT, ALB, ALT, and BCLC stage. Before PSM, the two groups showed a significant difference in Tibl level (> 23 µmol/L). After PSM, there were no significant differences in the Tibl level (> 23 µmol/L). In the triple therapy group, in one patient with PD, the PVTT was upstaged from Cheng’s type II to type III and progressed with several new nodule formations, which was considered unsuitable for surgery and treated with nonsurgical therapy with regorafenib.

**Table 1:** Preoperative patient demographics and tumor characteristics.

Variables	Before PSM (n = 100)			After PSM (n = 69)		
	Surgery alone (n = 76)	Triple therapy (n = 24)	P-value	Surgery alone (n = 23)	Triple therapy (n = 46)	P-value
Sex			0.919			1.000
Male	64	20		38	19	
Female	12	4		8	4	
Age (years)			0.364			0.864
≤ 60	49	13		25	12	
> 60	27	11		21	11	
HBsAg			0.436			0.448
Yes	65	22		39	21	
No	11	2		7	2	
Liver cirrhosis			0.294			1.000
Yes	63	22		42	21	
No	13	2		4	2	
Child-Pugh class			0.701			0.612
A	74	23		45	22	
B	2	1		1	1	
AFP			0.444			0.601
≤ 400 ng/mL	32	8		19	8	
> 400 ng/mL	44	16		27	15	
PIVKA-II			0.772			0.693
≤ 400 mAU/mL	18	5		12	5	
> 400 mAU/mL	58	19		34	18	
No. of tumor			0.652			0.392
Single	34	12		19	12	
Multiple	42	12		27	11	
Tumor diameter			0.685			0.579
≤ 10 cm	19	7		15	6	
> 10 cm	57	17		31	17	
ECOG PS			0.079			0.154
0	75	22		45	22	
1	1	2		1	1	
PVTT			0.736			0.490
Yes	41	12		18	11	
No	35	12		28	12	
Tbil			0.045			0.178
≤ 23 umol/L	69	18		40	17	
> 23 umol/L	7	6		6	6	
ALB			0.663			0.606
≤ 40 g/L	31	11		19	11	
> 40 g/L	45	13		27	12	
ALT			0.508			0.730
≤ 50 U/L	53	15		26	14	
> 50 U/L	23	9		20	9	
BCLC stage			0.939			0.673
A	14	5		9	5	
B	21	7		19	7	
C	41	12		18	11	
CNLC stage			0.956			0.842
Ib	14	5		9	5	
IIa	10	4		10	4	
IIb	11	3		9	3	
IIIa	41	12		18	11	

In the postoperative characteristics, the two groups showed a significant difference in MVI and R0 resection. There were no significant differences in the operative time and perioperative bleeding (**Table 2**).

**Table 2:** Postoperative and intraoperative clinicopathological features.

	Surgery alone (n = 76)	Triple therapy (n = 23)	P-value
MVI			<0.001
Positive	67	6	
Negative	9	17	
R0			0.017
Yes	50	21	
No	26	2	
operative time, min	245 ± 8.40	221 ± 9.93	0.067
intraoperative bleeding, ml	775 ± 126.03	523 ± 98.05	0.207

### Clinical responses and toxicity to neoadjuvant triple therapy (LEN + PD-1 + TACE)

**Figure 2:**
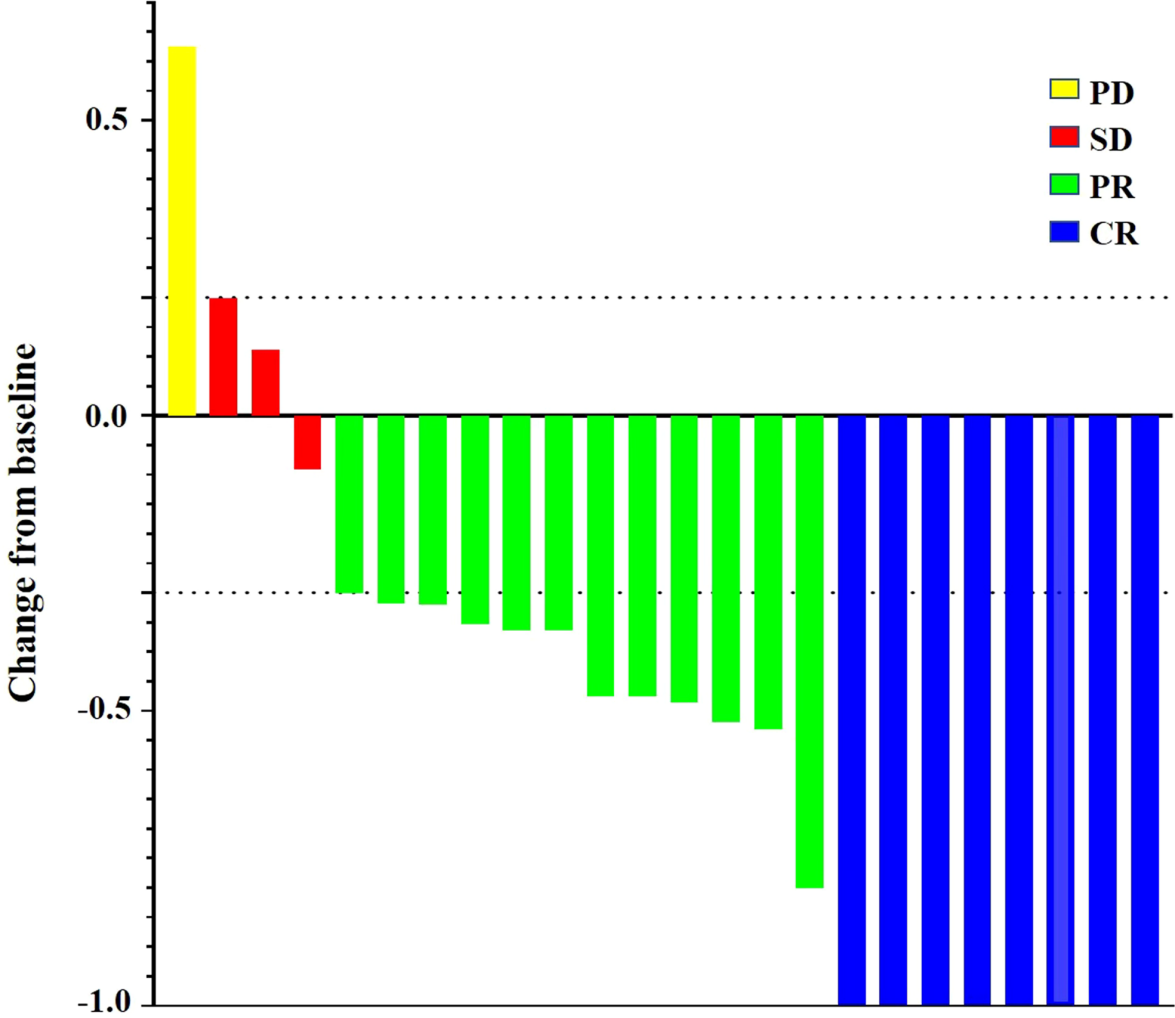
Percentage change from baseline in sums of maximum diameters of target lesions by the investigator using the mRECIST.

The mean waiting time for liver resection in triple therapy group was 4.1 months (range, 1.9–12.4 months). The median number of TACE procedures was two (range, 1–5). The ORR of neoadjuvant triple therapy was 83.33% (20 of 24) by the investigator, with CR in eight patients, PR in 12 patients, SD in three patients, and PD in one patient, while 79.17% (19 of 24) by BICR, with CR in eight patients, PR in 11 patients, SD in four patients, and PD in one patient (**Table 3**). For all patients with HCC who received neoadjuvant triple therapy, reductions in tumor size were reported in 87.5% (21 of 24) of patients with evaluable HCC by the investigator using mRECIST (**Figure 2**). Moreover, of the 23 patients who underwent successful SR, six had complete pathologic response, and four had major pathologic response. The treatment response of CR in imaging evaluated by BICR may not be PCR or MPR in pathology (**Supplementary Table 1** and **Supplementary Figure 1**). There may be tumor survival in patients with CR in imaging evaluated by BICR. The treatment response of PR in imaging evaluated by BICR may be MPR in pathology.

**Table 3:** Tumor Responses per Investigator and BICR Assessment.

Best Response, n (%)	Triple Therapy (n = 24)	
	Investigator	BICR
Complete response	8 (33.33%)	8 (33.33%)
Partial response	12 (50.00%)	11 (45.83%)
Stable disease	3 (12.50%)	4 (16.67%)
Progressive disease	1 (4.17%)	1 (4.17%)
Not evaluable	0	0
Objective response rate	20 (83.33%)	19 (79.17%)
Disease control rate	23 (95.83%)	23 (95.83%)

The treatment‐related adverse events (TRAEs) after neoadjuvant triple therapy are shown in **Table 4**. TRAEs occurred in 18 (75%) patients. The most common TRAEs were increased alanine aminotransferase level, decreased appetite, increased aspartate aminotransferase level, hypertension, hypothyroidism, diarrhea, increased blood bilirubin level, hand–foot skin reaction, decreased weight, and nausea and abdominal pain. None of the patients had grade 4 TRAEs, and all TRAEs were manageable after symptomatic therapy.

**Table 4:** Most common treatment-related AEs in the triple therapy group.

Preferred AE Term	Any Grade	Grade 1	Grade 2-3
Increased alanine aminotransferase	13	10	3
Hypertension	10	4	6
Hypothyroidism	10	5	5
Diarrhea	9	7	2
Increased blood bilirubin	8	8	0
Hand–foot skin reaction	7	4	3
Fatigue	7	6	1
Weight decreased	6	3	3
Nausea	4	4	0
Abdominal pain	4	2	2

### OS and DFS

**Figure 3:**
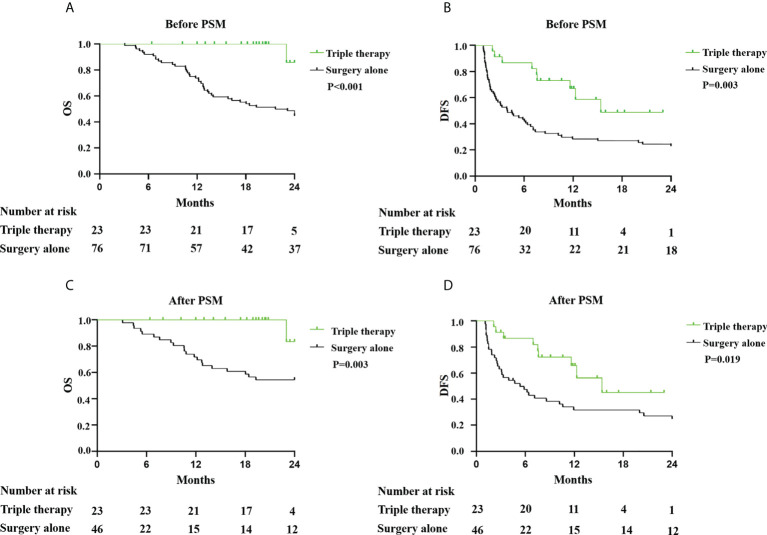
Kaplan–Meier analysis of OS and DFS in patients with HCC with high risk of recurrence treatment with triple therapy or surgery alone. **(A)** OS and **(B)** DFS in patients with HCC with and without triple therapy before PSM. **(C)** OS and **(D)** DFS in patients with HCC with and without triple therapy after PSM.

The median follow-up duration was 19.3 months (6.4–24.0 months) in the triple therapy group. The follow-up duration was 24 months in the surgery-alone group. The 6-, 12-, 18-, and 24-month OS rates were 100.0%, 100.0%, 100.0%, and 85.7%, respectively, in the triple therapy group but 92.1%, 73.7%, 53.9%, and 48.7%, respectively (P<0.001; **Figure 3A**). The 6-, 12-, 18-, and 24-month DFS rates were 82.2%, 66.95%, 48.8%, and 48.8% respectively, in the triple therapy group and 41.92%, 28.34%, 27.05%, 22.99%, and 13.3%, respectively, in the surgery-alone group (P=0.003; **Figure 3B**). Neoadjuvant triple therapy significantly increased both OS and DFS rates in resectable HCC with high risk of recurrence. The recurrence patterns are presented in **Table 5**. There was no significant difference in recurrence patterns between the two groups. After PSM, OS and DFS were significantly longer in the triple therapy group than that in the surgery-alone group (OS, *p*=0.003; DFS, *p*=0.019 **Figure 3C, D**). In addition, there was trend toward improvement in DFS for patients with pathological CR and MPR; however, there were not significant differences in DFS and OS between patients with pathological CR and MPR or without pathological CR and MPR (**Supplementary Figure 2**). The reason for this result may be that the number of cases is too small and the follow-up time is not long enough. We still think pathological CR and MPR have better postoperative survival than those without pathological CR and MPR.

**Table 5:** Recurrence location after SR.

	Surgery alone	Triple therapy	P-value
Recurrence location			0.408
Intrahepatic	41	8	
Extrahepatic	6	0	
Intrahepatic and Extrahepatic	13	1	

## Discussion

This study indicated that the combination of LEN + PD-1 + TACE as a neoadjuvant triple therapy in resectable HCC with high risk of recurrence improved outcomes. Neoadjuvant triple therapy before SR aimed to downstage HCC and reduce tumor burden instead of surgery alone in patients with HCC with extremely large tumors, multiple primary tumors, or major vascular invasion. In our study, triple therapy showed a high ORR (83.3%) with manageable toxicity in resectable HCC with high risk of recurrence. Six patients (26.1%) had pathological CR, and four patients (17.4%) had major pathological response. Neoadjuvant triple therapy significantly increased both the OS and DFS rates in resectable HCC with high risk of recurrence, compared with surgery alone before and after PSM. In addition, triple therapy reduces the rate of MVI and results in margin-negative resections.

To date, tumor size, primary tumor number, narrow-margin hepatectomy, and macrovascular invasion have been proven as risk factors for poor prognosis in HCC after SR^12,13^. Therapeutic strategies for patients with HCC with these high risks of recurrence remain controversial in the West and East (5–7). In Western guidelines, patients with HCC patients with high risk of recurrence were considered to have advanced BCLC stage B or C, and systemic therapy or TACE was recommended (5). In contrast, SR is more frequently performed in patients with HCC, which would provide survival benefit, in China and Southeast Asia if they met the criteria for liver function (6, 7). However, many studies have indicated that the postoperative prognosis of patients with HCC with macrovascular invasion, huge HCC (tumor size >10 cm), and multiple HCC tumors was poor (12, 13). Moreover, when HCC is adjacent to the major vascular structures (including the main portal branches, main trunks of the hepatic veins, and inferior vena cava), surgeons have to peel the tumor away from the vascular surface, leading to narrow-margin hepatectomy (RM <1 cm), and the surgical prognosis remains unsatisfactory due to a high risk of recurrence (27). Therefore, patients with advanced HCC may still be a controversial indication for SR.

To solve these problems, postoperative adjuvant or preoperative neoadjuvant therapy has been advocated to improve the postoperative prognosis of patients with HCC at high risk of recurrence. Numerous clinical trials have shown that the use of combination TKIs and ICIs has become a new standard of care option for uHCC (21–23). As for preoperative neoadjuvant therapy, in a single-arm phase 1b study, Ho indicated that the combination of cabozantinib and nivolumab in patients with HCC with borderline or locally advanced HCC as neoadjuvant therapy followed by SR is feasible and can result in margin-negative resections (17). A single-arm, open-label, phase 2 trial showed that neoadjuvant anti-PD-1 monotherapy in resectable HCC before SR resulted in a high rate of tumor pathological responses (28). Given the recent approval of several systemic therapeutic agents for HCC, it is possible that neoadjuvant therapy before SR in resectable HCC would improve surgical outcomes.

TACE is the standard treatment for intermediate-stage HCC (29). Studies have also indicated that TACE plus other treatment modalities, such as local ablation, radiation therapy, or systemic therapy, have been actively conducted and benefits patients with HCC (30, 31). Based on these findings, in our previous study, we showed that triple therapy in uHCC achieved a satisfactory ORR and was converted to resection^24^. Therefore, we assessed the effect of triple therapy, which was used as neoadjuvant therapy, in the treatment of resectable HCC with high risk of recurrence. Our results showed that triple therapy achieved a high ORR and significantly increased both the OS and DFS rates in resectable HCC with high risk of recurrence, compared with surgery alone. In our study, we also found that the triple preoperative neoadjuvant therapy reduced tumor size and MVI rate and improved R0 resection rate in HCC with high risk of recurrence, which would improve the prognosis. These confirms that the tumor is sensitive to the preoperative neoadjuvant therapy. Therefore, triple therapy may play a potential role in neoadjuvant therapy in the treatment of resectable HCC with a high risk of recurrence. However, more evidence needs to be accumulated.

Studies have shown that combination therapy with different treatment modalities may improve outcomes in HCC (24, 30). However, the mechanisms by which these modalities affect one another remain unclear. In triple therapy, PD-1 blocks PD-L1 to its receptor on T cells to suppress the proliferation and effector function of T cells to inhibit tumor growth (32, 33). However, it was not sufficient to initiate adequate levels of anticancer immunity in HCC *via* the PD-L1/PD-1 axis blockade alone (32, 33). TACE would enhance the clinical efficacy of PD-1 antibodies by activating the release of tumor-specific antigens (34). Unfortunately, TACE also creates a hypoxic microenvironment and activates the release of HIF-1 alpha, vascular endothelial growth factor, and fibroblast growth factor, leading to tumor angiogenesis and progression (34). LEN, a multikinase inhibitor of VEGF receptors 1-3, FGF receptors 1-4, platelet-derived growth factor receptor-a, RET, and KIT, could suppress tumor angiogenesis and antitumor immunity in tumor microenvironments, which would enhance the effect of PD-1 antibodies and TACE (30, 34). This is probably why triple therapy was associated with a high rate of tumor responses and was effective in improving the prognosis of patients with HCC with high risk of recurrence. However, further studies are required to elucidate the mechanisms of triple therapy.

This study had several limitations. First, this study had a retrospective design. Second, the number of patients with HCC treated with triple therapy (LEN+PD-1+TACE) is small. Third, surgical treatment and postoperative management were performed by different clinicians from different centers, which may have affected the surgical outcomes. Finally, the generalizability of our results may be limited because HCC in our study had a high proportion of HBV. Further prospective study should seek to resolve these issues.

## Conclusion

In conclusion, neoadjuvant combination therapy with LEN+PD-1+TACE (triple therapy) before SR is associated with a high rate of tumor responses and is effective in improving the prognosis of patients with HCC with high risk of recurrence.

## Data availability statement

The raw data supporting the conclusions of this article will be made available by the authors, without undue reservation.

## Ethics statement

The studies involving human participants were reviewed and approved by the research ethics committee of Fujian Provincial Hospital. The patients/participants provided their written informed consent to participate in this study.

## Author contributions

Conceived and designed the research: M-LY and Ju-YW. Data acquisition: Ju-YW, Ji-YW, Y-NL, F-NQ, and S-QZ. data analysis: Ju-YW, Ji-YW, Z-YY, Y-FC, and BL. Drafting the manuscript: Ju-YW and M-LY. All authors read and approved the final manuscript.

## Funding

This research was supported by Startup Fund for scientific research, Fujian Medical University (Grant number: 2018QH1112).

## Conflict of interest

The authors declare that the research was conducted in the absence of any commercial or financial relationships that could be construed as a potential conflict of interest.

## Publisher’s note

All claims expressed in this article are solely those of the authors and do not necessarily represent those of their affiliated organizations, or those of the publisher, the editors and the reviewers. Any product that may be evaluated in this article, or claim that may be made by its manufacturer, is not guaranteed or endorsed by the publisher.

## Supplementary material

Supplementary Figure 1 The corresponding IHC pictures and images in patients treated with triple therapy. **(A)** The treatment response of CR in imaging evaluated by BICR and PCR in pathology. **(B)** The treatment response of CR in imaging evaluated by BICR and not PCR or MPR in pathology. **(C)** The treatment response of PR in imaging evaluated by BICR and MPR in pathology.

Supplementary Figure 2 Kaplan–Meier analysis of OS and DFS in patients with PCR or MPR and without PCR or MPR in triple therapy group. **(A)** OS and **(B)** DFS in patients with patients with PCR or MPR and without PCR or MPR in triple therapy group.

## Abbreviations

HCC, Hepatocellular carcinoma; SR, surgical resection; LEN, lenvatinib; PD-1, anti-PD-1 antibodies; TACE, transcatheter arterial chemoembolization; PSM, Propensity score matching; BCLC, Barcelona Clinic Liver Cancer; PVTT, portal vein tumor thrombus; TKIs, tyrosine kinase inhibitors; ICIs, immune checkpoint inhibitors; CT, computerized tomography; MRI, magnetic resonance imaging; mRECIST, modified criteria; CR, complete response; PR, partial response; SD, stable disease; PD, progressive disease; ORR, objective response rate; OS, overall survival; DFS, disease-free survival; PIVKA-II, vitamin K absence-II; HBsAg, hepatitis B surface antigen; MVI, microvascular invasion; AFP, α-fetoprotein; Tbil, total bilirubin; ALB, albumin; ALT, alanine aminotransferase; TRAEs, treatment‐related adverse events.
