# Supplementary figures and images for: Lenvatinib combined with anti-PD-1 antibodies plus transcatheter arterial chemoembolization for neoadjuvant treatment of resectable hepatocellular carcinoma with high risk of recurrence: A multicenter retrospective study
Source: Front Oncol. 2022 Sep 21;12:985380. doi: 10.3389/fonc.2022.985380 (PMC9534527; doi:10.3389/fonc.2022.985380)

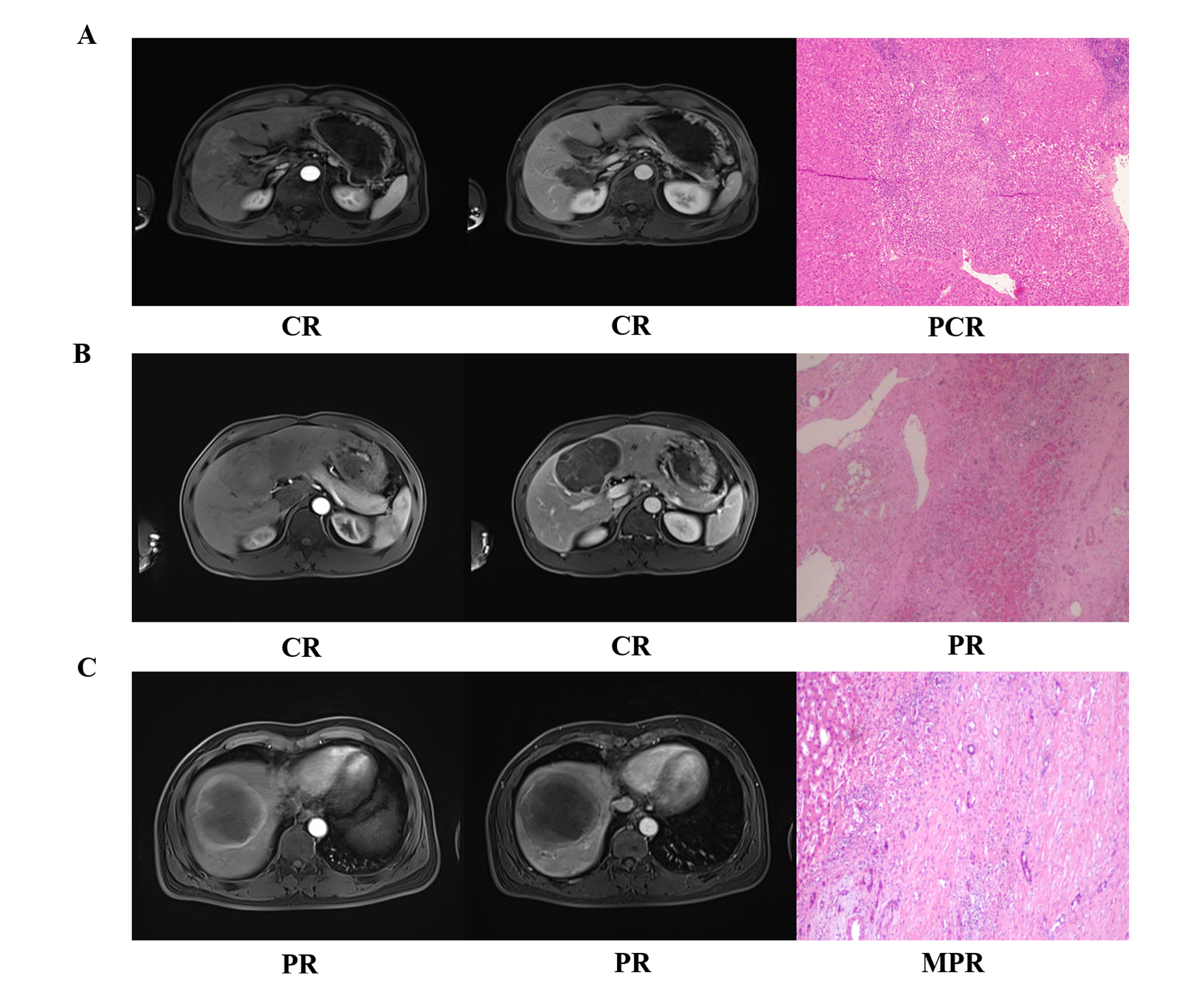

Supplement: Supplementary Figure 1 — The corresponding IHC pictures and images in patients treated with triple therapy. (A) The treatment response of CR in imaging evaluated by BICR and PCR in pathology. (B) The treatment response of CR in imaging evaluated by BICR and not PCR or MPR in pathology. (C) The treatment response of PR in imaging evaluated by BICR and MPR in pathology. [file Image_1.jpeg]

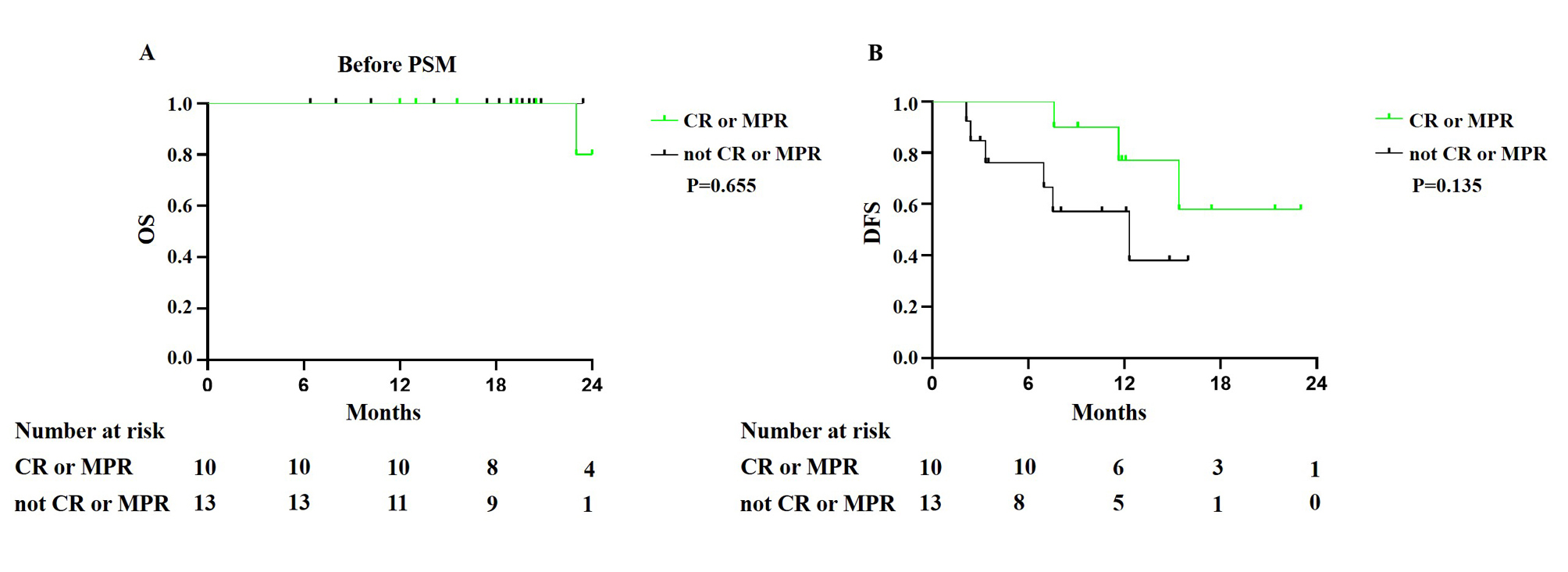

Supplement: Supplementary Figure 2 — Kaplan–Meier analysis of OS and DFS in patients with PCR or MPR and without PCR or MPR in triple therapy group. (A) OS and (B) DFS in patients with patients with PCR or MPR and without PCR or MPR in triple therapy group. [file Image_2.jpeg]
