# Supplementary material for: Lenvatinib combined with anti-PD-1 antibodies plus transcatheter arterial chemoembolization for neoadjuvant treatment of resectable hepatocellular carcinoma with high risk of recurrence: A multicenter retrospective study
Source: Front Oncol. 2022 Sep 21;12:985380. doi: 10.3389/fonc.2022.985380 (PMC9534527; doi:10.3389/fonc.2022.985380)
Supplement: Supplementary file 3 [file Table_1.docx]

**Table 1. The differences between treatment response in imaging evaluated by BICR and pathology.**

| **BICR**  **Pathology** | **CR** | **PR or SD** |
| --- | --- | --- |
| **PCR** | **6** | **0** |
| **MPR** | **2** | **2** |
| **Not PCR or MPR** | **0** | **13** |
